# Diffusion as a Natural Contrast in MR Imaging of Peripheral Artery Disease (PAD) Tissue Changes. A Case Study of the Clinical Application of DTI for a Patient with Chronic Calf Muscles Ischemia

**DOI:** 10.3390/diagnostics11010092

**Published:** 2021-01-08

**Authors:** Weronika Mazur, Małgorzata Urbańczyk-Zawadzka, Robert Banyś, Rafał Obuchowicz, Mariusz Trystuła, Artur T. Krzyżak

**Affiliations:** 1 Faculty of Geology, Geophysics and Environmental Protection, AGH University of Science and Technology, Mickiewicza Avenue 30, 30-059 Cracow, Poland; Weronika.Mazur@fis.agh.edu.pl; 2 Faculty of Physics and Applied Computer Science, AGH University of Science and Technology, Mickiewicza Avenue 30, 30-059 Cracow, Poland; 3 Department of Radiology and Diagnostic Imaging, John Paul II Hospital, Prądnicka Street 80, 31-202 Cracow, Poland; gosia_urbanczyk@poczta.onet.pl (M.U.-Z.); p.banys@szpitaljp2.krakow.pl (R.B.); 4 Department of Diagnostic Imaging, Jagiellonian University Medical College, Jakubowskiego 2, 30-688 Cracow, Poland; rafalobuchowicz@su.krakow.pl; 5 Department of Vascular Surgery with Endovascular Procedures Subdivision, John Paul II Hospital, Prądnicka Street 80, 31-202 Cracow, Poland; m.trystula@szpitaljp2.krakow.pl

**Keywords:** diffusion tensor imaging, peripheral artery disease, diffusion tensor tractography, BSD calibration

## Abstract

This paper reports a first application of diffusion tensor imaging with corrections by using the B-matrix spatial distribution method (BSD-DTI) for peripheral artery disease (PAD) detected in the changes of diffusion tensor parameters (DTPs). A 76-year-old male was diagnosed as having PAD, since he demonstrated in angiographic images of lower legs severe arterial stenosis and the presence of lateral and peripheral circulation and assigned to the double-blind RCT using mesenchymal stem cells (MSCs) or placebo for the regenerative treatment of implications of ischemic diseases. In order to indicate changes in diffusivity in calf muscles in comparison to a healthy control, a DTI methodology was developed. The main advantage of the applied protocol was decreased scanning time, which was achieved by reducing b-value and number of scans (to 1), while maintaining minimal number of diffusion gradient directions and high resolution. This was possible due to calibration via the BSD method, which reduced systematic errors and allowed quantitative analysis. In the course of PAD, diffusivities were elevated across the calf muscles in posterior compartment and lost their anisotropy. Different character was noticed for anterior compartment, in which diffusivities along and across muscles were decreased without a significant loss of anisotropy. After the intervention involving a series of injections, the improvement of DTPs and tractography was visible, but can be assigned neither to MSCs nor placebo before unblinding.

## 1. Introduction

Peripheral arterial disease (PAD) is a condition in which blood supplied to peripheral tissues arteries is obstructed, which leads to ischemia of these tissues. In the chronically progressive deficit in oxygenated blood inflow in the course of PAD, the muscle tissue that uses the most oxygen during its work is the first to manifest this clinically. While walking, pain in the calf muscles occurs; the greater the restriction of the inflow, i.e., the restriction of oxygen supply, the faster it occurs. In extreme cases, if we do not improve blood flow, all tissues, including muscles, are necrotized.

The main diagnostic methods before qualifying the patient for revascularization are ultrasound examination with color imaging, angiography or computed tomography angiography. In patients with critical ischemia of the lower extremities, in whom revascularization is not possible, in addition to imaging small yet patent arteries, the assessment of blood supply to the muscles of the lower extremities may be helpful.

Healthy muscles have an anisotropic structure. Changes in the muscle tissue caused by ischemia lead to gradual degradation of cell structures (including protein denaturation), cytoplasm swelling, and disintegration of the cell membrane, and thus disrupt the anisotropic structure of the muscles. As magnetic resonance (MR) diffusion tensor imaging (DTI) is very sensitive to microgeometry, it can be used to detect muscle structural changes caused by ischemia. So far, DTI has been used to detect nervous system injuries [1], in skeletal muscle injuries [2,3], heart muscle injuries [4,5] and in innervation disorders [6,7,8]. It has also been shown that the second (λ_2_) and third (λ_3_) eigenvalues reflect the size of the endomysium and myofiber, respectively [9,10,11].

Stem cells used in regenerative medicine are the primary cells of the body that have the ability to multiply and transform into various, specialized types of daughter cells. These, in turn, can become the starting material for damaged tissue or organ. They are used for the regeneration of nerves, joints, skin, heart muscle and skeletal muscles by acting directly on damaged tissues and indirectly by stimulating the formation of collateral microcirculation (neo-angiogenesis), which in turn improves blood supply to the muscles and allows regeneration.

In this case study, DTI is used to examine the calf muscles of a patient with PAD who have received intraarterial and intramuscular injections of CardioCell based on mesenchymal stem cells (MSCs) or placebo which was produced according to GMP rules at PBTiK UJ CM (license no. 145/0323/15), in a double-blind RCT. The aim of this report is twofold: to outline the development of the approach for the non-invasive diagnosis of PAD by DTI and to monitor the condition of the calf muscles after the administration of therapeutic injections by detecting changes of the diffusion tensor parameters (DTPs) and tractography.

## 2. Case Study

The diagnostic approach described in the next section was tested on a patient selected randomly from a group of 55 patients from N-O CLI clinical trial, registered under EudraCT No. 2016-004684-40, conducted in accordance with GCP requirements. A 76-year-old male was referred to the Outpatient Clinic of the Vascular Surgery Department of the John Paul II Hospital in Cracow, due to critical ischemia of the right lower limb. This condition was caused by atherosclerosis, diabetes mellitus and possibly also immune responses in the course of rheumatoid arthritis. The patient was a heavy smoker in the past. These conditions had caused the build-up of atherosclerotic plaques in the arteries, causing a state of ischemia in many organs, but especially in his right lower limb. The symptom of such a critical supply to the leg tissues of oxygen carried by the blood is pain, in the first place in the muscles, not only during exercise, i.e., while walking, but even at rest. The extreme stage of ischemia is tissue necrosis, and when the inflow of oxygenated blood is not improved, necrotic changes require limb amputation.

During the last few years, this patient was operated upon many times, both by endovascular methods (arterial recanalization with angioplasty and stent implantation), and by performing open surgery (endarterectomy and by-passes). Due to the exhaustion of all possibilities of revascularization, he was offered an experimental method of treatment using mesenchymal cells (so-called stem cells) in a double-blind RCT. These cells were isolated from Wharton’s jelly of neonatal umbilicals. In this program, research was focused on creating a new network in arterial microcirculation through stimulation with stem cells. As a result, it would improve the blood supply to the limb and save it from amputation.

The intraarterial and intramuscular injections in a double-blind RCT intervention was initiated following the first DTI examination (E1). Therapy encompassed three injections every seven weeks and after the whole series (83 days after E1) the DTI examination was repeated (E2; first follow-up). One healthy volunteer was enrolled in the study (control). The study protocol was designed according to the guidelines of the Declaration of Helsinki and Good Clinical Practice standards and conducted at the Vascular Surgery Department with Endovascular Procedures Subdivision, John Paul II hospital in Cracow, Poland. The Institutional Ethical Committee on Human Research approved the studies and the publication of anonymized medical images. Informed consent was also collected from the patient.

## 3. DTI Procedure Developed for the Diagnosis and Intervention Monitoring

Both DTI examinations were performed on a 3T MR system (Siemens Skyra 3 T, Erlangen, Germany) with the application of an eight-channel TORSO body coil. Lower legs were examined axially in terms of T_1_-weighted MR images (repetition time, TR = 440 ms, echo time, TE = 10.8 ms) and T_2_-weighted MR images (TR = 3800, TE = 70 ms) with fat suppression by using a Fast Spin Echo (FSE) sequence. The images were primarily used to depict the anatomical structures and detect muscle edema. DTI were acquired using the Echo Planar Imaging (EPI) sequence with six diffusion gradient directions. In the DTI protocol b-value = 350·10^3^ s/mm^2^, TR/TE, 5200/64 ms, FOV = 59 × 39 cm^2^, Number of Scans, NoS = 1, 384 × 300 Px matrix; slice thickness was equal to 8 mm, while no interleaved slices were assured. Each DTI acquisition lasted about 2.5 min.

Additionally, since the analysis is quantitative, the B-matrix spatial distribution (BSD) method was applied in order to eliminate the systematic errors in DTI [12,13]. The approach relies on the determination of real B–matrices on a voxel-by-voxel basis with the application of anisotropic phantoms. After the BSD calibration of a gradient field, the diffusion tensor can be more accurately determined, while the bias due to systematic errors is eliminated [14]. The application of this method is especially important in fiber tracking, in which fiber tracts are more reliably determined, and changes can be better detected [15].

Images were analyzed in terms of mean diffusion tensor parameters (DTPs) and fiber tracking using the in-house BSD-DTI software (BSD-DTI ver. 2.0, AGH UST, Cracow, Poland). DTPs analyzed in the study were fractional anisotropy (FA), mean diffusivity (MD) and three eigenvalues (λ_1_, λ_2_, λ_3_). ROIs were selected in the regions of three muscles: Gastrocnemius Medialis (GM), Soleus (SOL) and Tibialis Anterior (TA), which were identified based on T_1_-weighted images. ROIs were circles with a radius consisting of four pixels.

Fiber tracking was performed by applying seeding ROIs (with each seed step equal to 1 voxel) allowed for bidirectional tracking with the integration step set to 0.1 voxel. The seeding ROIs were drawn based on the T_1_-weighted images in the SOL muscle. FA range was equal to 0.15-1, and an angle change was set to be smaller than 45 per integration step. In the images, seven slices were subjected to analysis, in which the seeding ROIs were circles having a radius equal to eight pixels. The location of the ROI was chosen on the basis of cuts made on a frozen cadaver (Visible Human Project U.S National Library of Medicine). The obtained fiber tracts were analyzed qualitatively and quantitatively via the fiber tracts density (FTD) parameter. This is calculated as the ratio of the number of tracts per volume of voxels in a cylinder obtained from stacking ROIs from all slices.

## 4. Results and Discussion

**Figure 1:**
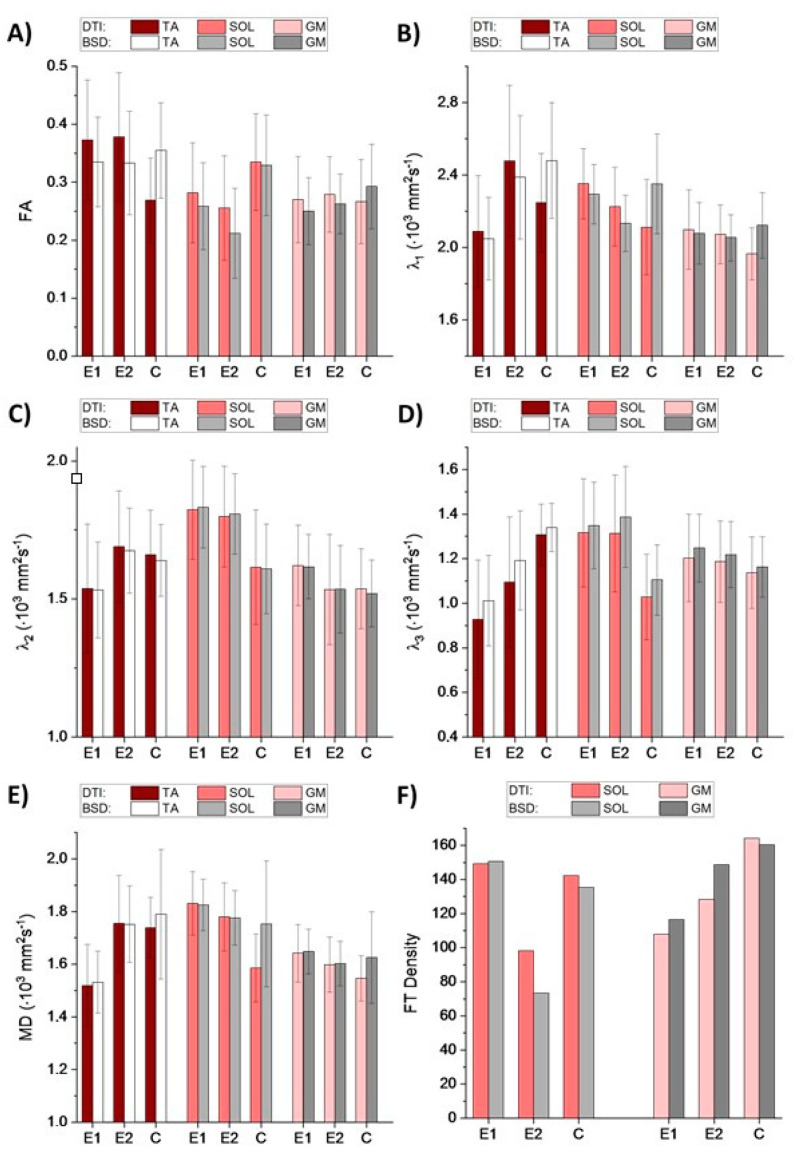
Diffusion tensor parameters: fractional anisotropy (FA) (**A**), λ_1_ (**B**), λ_2_ (**C**), λ_3_ (**D**), mean diffusivity (MD) (**E**) and fiber tracts (FT) density (**F**) obtained for the patient before (E1) and after (E2) the intervention and the control (C) with and without the calibration of a gradient field using the B-matrix spatial distribution (BSD) method.

The BSD-DTI method eliminates systematic errors present during the calculation of a diffusion tensor. The method relies on the Generalized Stejskal-Tanner equation; the importance of the application to nervous system diagnostics based on diffusion tensor tractography was pointed out recently [12,13,14,15,16,17,18]. The effectiveness of even the lean approach was also shown (in relation to the BSD method), where the effective value of the B-matrix was calibrated on the basis of isotropic phantom measurements [19]. There is also an alternative approach to correct the DTI images based on the knowledge of the coil tensor, L, obtained from the manufacturer or experimentally [20]. Currently, however, it is not possible to apply this approach in practice and there is no clear evidence of its effectiveness (the impact of factors other than heterogeneity of the gradient coils on the distribution of gradient fields for different diffusion sequences). In our study, the observed effect of the calibration using the BSD method is not the same for all of the subjects. It can be seen in Figure 1, that FA and λ_1_ are significantly underestimated for control, while overestimated for the patient when calculated from the uncalibrated tensor. Considering that the differences determining muscle status can be subtle, the application of BSD-DTI seems essential for the use of diffusion as a disease marker.

**Figure 2:**
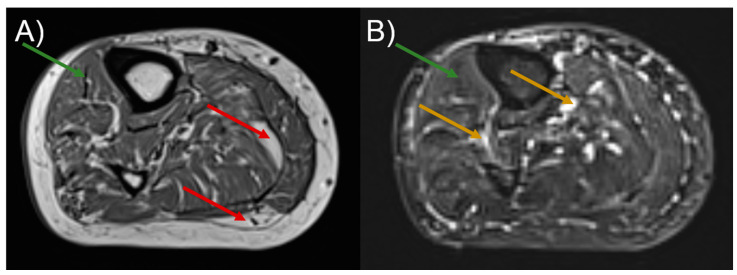
T_1_-weighted (**A**) and T_2_-weighted (**B**) images of the patient’s calf acquired before the intervention (following the E1 diffusion tensor imaging (DTI) examination). Red, green and yellow arrows indicate fatty accumulation in muscles, fibrosis and blood vessels, respectively.

As shown in the Section 1, DTPs can reflect muscle status. Increased MD may indicate broadened spaces within an analyzed volume of interest. Increased λ_2_ and λ_3_ indicate elevated diffusivity across the muscle’s long axis, which means that the cross-sectional areas of the muscle and endomysium are extended. In the case of PAD, it is expected that fatty accumulation, newly-formed vessels (collateral circulation), fibrosis and undernourished muscles (see Figure 2) will cause changes in the abovementioned parameters. These changes are clearly discernible in Figure 1. In the diseased muscles, the partial loss of anisotropy compared to the healthy ones can be seen, especially in the SOL (Figure 1A). Transversal diffusivity (reflected in λ_2_ and λ_3_) in SOL is increased, possibly due to fatty accumulation. Longitudinal diffusivity (λ_1_) is very similar to the healthy muscle, which may be caused by the presence of multiple blood vessels, that can increase diffusion coefficient in the direction of a blood flow. In GM muscle, differences in diffusivities correspond to SOL, but they are more subtle due to fewer blood vessels and lower fatty infiltration. In the anterior compartment the character of changes is different than in the posterior one. In TA muscle all diffusivities (λ_1_, λ_2_, λ_3_ and MD) are decreased, with FA almost not affected. This may result from fibrosis in the muscle (Figure 2). It was hypothesized by Sanz-Requena et al. [21] that the fat content in lower leg muscles would cause a decrease of apparent diffusion coefficient. Therefore, decrease of diffusivities in the patient’s TA muscle may also indicate its dehydration.

**Figure 3:**
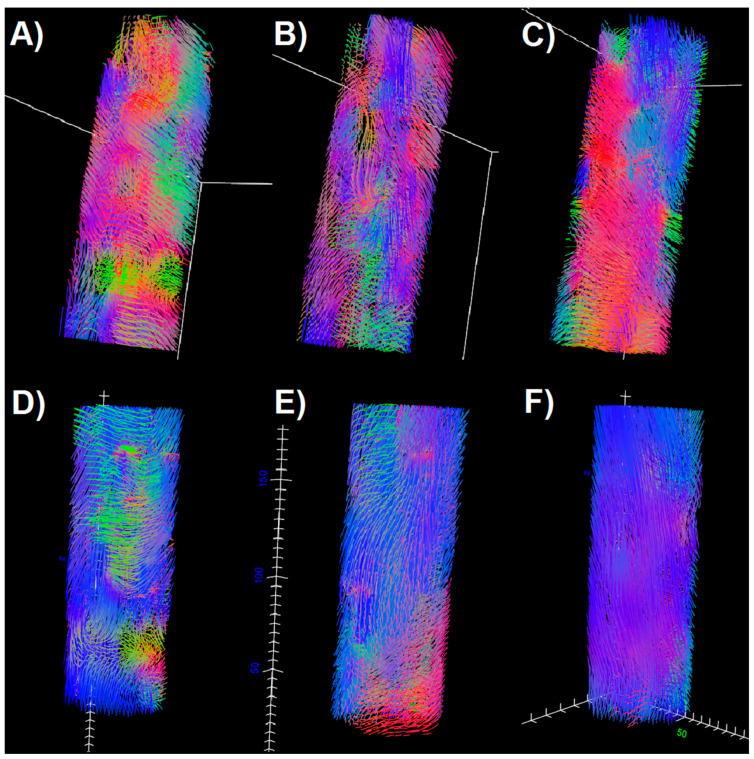
Fiber tracts obtained for the soleus (SOL) (upper row) and gastrocnemius medialis (GM) (lower row) muscles for the patient in the first examination (**A**,**D**), for the patient after the intervention (**B**,**E**) and for the healthy control (**C**,**F**).

Comparing E1 and E2 examinations with the control, it can be seen that in TA and GM the DTPs from E2 become closer to the values obtained for the control. In SOL, the differences are higher after the intervention. However, fiber tracts (Figure 3) evince improvement in terms of the fiber tract directions in both SOL and GM. It is clearly visible that tractography for E2 contains more vertical (blue), organized fibers than for E1, and becomes more similar to the tractography for healthy legs. The density of fibers is only improved for GM (Figure 1F and Figure 3). However, considering that this paper is based on the preliminary results from an ongoing project, it is as yet unknown whether these results reflect the patient’s actual condition.

## 5. Conclusions

In this paper, diffusion was proposed as a natural marker of skeletal muscle condition in PAD. Diffusion tensor parameters and tractography after the correction by using the BSD method were compared for examinations conducted for patient before and after the injections in the double-blind RCT, and with a healthy control. This case study shows that in the course of PAD, both anterior and posterior compartments are distinguished by the change of DTPs in comparison to control, while the character of these changes is compartment-dependent. Moreover, diffusivity and anisotropy changes are different for the muscles containing fibrosis, fatty accumulation, and depend on the degree of the angiogenesis. After injections, a very good improvement was observed for DTPs in the TA muscle, and a discernible improvement was seen in the GM muscle. In the SOL, the differences between the patient and the control increased in the follow-up examination. In terms of fiber tracking, the tracts in E2 were more similar to the control in terms of directions (SOL, GM) and density (GM). This may indicate the progression of muscle anisotropy and structure, respectively. The progression will be assigned to the MSCs therapy or placebo after the unblinding.

## Data Availability

The data presented in this study are available on request from the corresponding author.
